# Inflammation Thread Runs across Medical Laboratory Specialities

**DOI:** 10.1155/2016/4121837

**Published:** 2016-07-14

**Authors:** Urs Nydegger, Thomas Lung, Lorenz Risch, Martin Risch, Pedro Medina Escobar, Thomas Bodmer

**Affiliations:** Labormedizinisches Zentrum Dr. Risch and Kantonsspital Graubünden, 7000 Chur, Switzerland

## Abstract

We work on the assumption that four major specialities or sectors of medical laboratory assays, comprising clinical chemistry, haematology, immunology, and microbiology, embraced by genome sequencing techniques, are routinely in use. Medical laboratory markers for inflammation serve as model: they are allotted to most fields of medical lab assays including genomics. Incessant coding of assays aligns each of them in the long lists of big data. As exemplified with the complement gene family, containing C2, C3, C8A, C8B, CFH, CFI, and ITGB2, heritability patterns/risk factors associated with diseases with genetic glitch of complement components are unfolding. The C4 component serum levels depend on sufficient vitamin D whilst low vitamin D is inversely related to IgG1, IgA, and C3 linking vitamin sufficiency to innate immunity. Whole genome sequencing of microbial organisms may distinguish virulent from nonvirulent and antibiotic resistant from nonresistant varieties of the same species and thus can be listed in personal big data banks including microbiological pathology; the big data warehouse continues to grow.

## 1. Introduction

Biologic inflammation in its multifaceted subsistence lends itself to delineation by big laboratory data IT—in this age of data warehouse extension. Many life maintaining biological interactions function as multicomponent weight balance, equilibrium standing for health. Settling on one or the other side to vacate fine adjustment may go on to result in final targeting be it for health maintenance or to develop pathological transformation. The paths to excess are paved with stop- and go-signaling: activation signals can be held back with the hazard to cause overshoot in the other direction of the balance. To such intrinsically complex regulatory framework of a single system adds up the interaction between different systems involving health maintaining cross-reactivities or expanding pathological effects.

Thus a large amount of metabolites, proteins, intermediate and terminal enzymes interact simultaneously to maintain physiological wellbeing or they will thwart equilibrium. When attempting diagnosis, medical laboratories test for single disease-related leading analytes/markers and they go for an appropriate choice to give patients and physicians a representative picture to tailor therapy. We here describe some approaches to sort out the relevant results for patient care in precision medicine. In this analysis we exclude the doctors' choice on categories on order sheets of the appropriate lab assays at the outset, assuming that big data accumulate over time during medical checkups unrelated to a single morbus.

## 2. Metabotyping May Circumscribe Inflammation in the Lab

**Figure 1:**
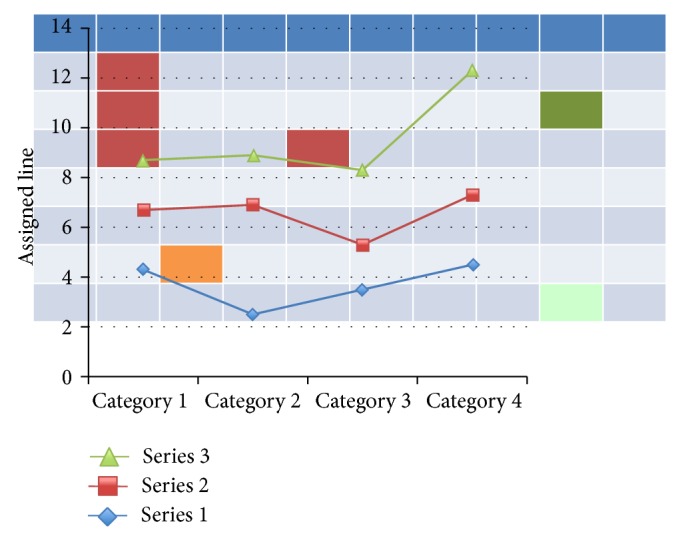
Metabolome profiling. Real-time metabolome profiling by injection of living bacteria, yeast, or mammalian cells into a high-resolution mass spectrometer enabling automated monitoring of several hundred metabolites can be simultaneously quantitatively estimated within minutes in a circuitry displayed in the work published by the Swiss Federal Institute of Technology. Output-fluxes of ~300 compounds using automated monitoring in 15–30 s cycles over several hours are possible. The figure is a simplified transposition of single data points from hundreds of possible analyses (squares, randomly highlighted with colors) becoming linked using bioinformatics into series and categories meaningful for exploration [29].

Metabotyping and high-resolution omics data has the promise to picture diseases based on metabolite's profile or ratios of selected analytes and might develop into a relevant component of diagnosis and treatment of single nosological entities. Mechatronic engineering designs, testing and operation of machinery and equipment, in which there is a high level of functional integration of mechanical systems with electronics and computer control in laboratory equipment brings information gain from metabotyping upfront. Biologists of the Swiss Federal Institute of Technology work on real-time simultaneous analysis of hundreds of analytes measured with the same instrument [1], results of which might be funneled into biocomputing circuits. Mathematical models are then used to quantitatively relate metabolomics, expression, and proteomics data to the functional network output related to fluxes (Figure 1). The usability of such waves of IT based information, particularly if used in health-monitoring systems, will need original/innovative approaches for secure storage [2].

We here attempt to envision the inflammation portion of the whole body metabolism as an envelope containing interactive signal circuits which interact at the frontend of genetic, transcriptional, and proteomic backgrounds and react to inflammation inducing forces: the current view of senescence being brought forward, at least in part, by inflammatory mechanisms has coined the neologistic term of inflammaging, none the least of these being senescence (inflammaging) [3–6]. Our update can be read as a background to discern digital memories eventually leading to biological computer science [7].

## 3. Medical Laboratory Copes with Big Data

Each single patient produces data in the long run with her or his standard data entry description: accession number, sample number, patient ID, sex, birthday, clinic, ward, doctor, order comment collection source, (repeat) collection date, and sample comment. Generation of big data cannot be circumvented since long. Dialog boxes upon receipt of samples in the lab may help to reduce data flow but cannot ban it. Analysis of most disease-related metabolites, including glycoproteidic biomarkers for disease diagnosis, is based on ELISA, ECLIA, and enzyme-substrate colorimetric/light extinction/electrochemoluminescence (ECL) technology.

Mass spectrometry with improved resolution is now often preceded by multidimensional chromatographic separation schemes which enlarges the spectrum of possible analytes. A uniform distribution of the number of acquired MS/MS, protein, and peptide identifications undergo proteomic runs which allow protein identification on large scales estimated up to as high as ~14 000 proteins and ~250 000 unique peptides [8].

A large spectrum glycoprotein profiling in plasma, serum, other bodily fluids or tissues is thus possible. Transplantation of donor organs into patients in need has exceeded HLA-compatibility requirements since ABO blood type system incompatible donor/recipient pairs are becoming routine [9]. Infectious and inflammatory complications remain within limits in such settings and their prophylaxis includes tight lab test controls. Subclinical inflammation [10], reliably diagnosed using C-reactive protein (CRP) serum level cutoff ≥ 10 mg/L, constitutes a risk factor for the development of interstitial fibrosis and seems to reflect not only inflammation but general well being: CRP is a subtle separator for hand grip strength, physical performance, and decline in older populations [11].

Serum pattern recognition compounds, among them CRP, bind to apoptotic cells and nucleoprotein autoantigens and Fc*γ* receptors to ultimately inhibit plasmacytoid dendritic cell interferon responses which are elicited by autoantibody immune complexes [12]. The monomeric form of CRP ALS inhibits renal cell-directed complement activation mediated by properdin [13]. Transplantation medicine largely contributing to big data is going to include ABO-glycan microarray results which now allow detailed characterization of donor-specific antibodies necessary for effective transplant management of solid organs [14–16].

**Figure 2:**
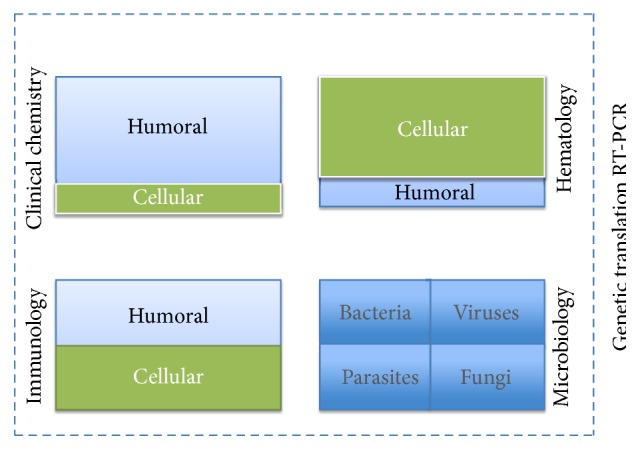
The four main specialities of laboratory medical analyses. The four major sections of medical laboratory analyses are shown using a four-box pattern, that is, clinical chemistry, haematology, immunology, and microbiology. Approximate quota of humoral and cellular assays is given. The big data data warehouse is substantially enlarged if each assay is completed/translated using DNA testing by real-time polymerase chain reaction (RT-PCR). Genetic embracement is drawn as broken line.

In health care, the use of “big data,” meant to use the large number of digitalized single analyses accumulated daily [17], a computational medicine, that is, eHealth or Electronic Health or Medical Record (HER and EMR), expands at the expense of health care professionals to read its message. Data warehouses containing big data need continuous validation, data management for updates, and most importantly an analysis to convert such resource into clinically relevant information for medical care [18]. Mass spectrometry and bioinformatics IT solutions may establish profiles which help to diagnose frequent and rare disease alike. The designation of given lab analyses as biomarker is currently moving towards genome sequencing to reveal genetic risks for future illness (Figure 2) [19]—obviously a component of personal big data; patients on their own will thus access to their data bank. Multibiomarker diagnosis and disease activity scoring, often a difficult curse to work, is being bundled by the clinician intending to focus on patients' complaint and clinical signs and symptoms pattern clustered together by cross-reactive algorithms [20, 21].

## 4. An Attempt to Categorize

For practical reason, we here lend the four major fields of laboratory medicine in Switzerland, under the auspices of a federal organization, Foederatio Medicorum Analyticorum Helveticorum (FAMH): clinical chemistry, immunology, haematology, and microbiology with genetics wrapping up each of these (Figure 2). To a large extent, this distinction of laboratory analyses by category corresponds to international habits defined by the International Federation of Clinical Chemistry (IFCC). The substantial expansion of the analyses performed boosts each of these specialities alike, enriching the respective fields with unprecedented wealth of data. This brings medical doctors to dilemmas on the appropriate choice of information for patient care—in the present context focusing on inflammation. To turn big data into appropriate data has become a topic of many fields endowed with data warehouses. Thus computational methods may throw a bridge from bench to bedside and vice versa [7, 25] and web-based platforms would allow seamless interaction between warehouse and patient-centered information as recently put forward through an EU project [26]. Academic as well as business intelligence literature counsels are available for assisting transformation of big data into a selection of data assembled for care of single disease entities (http://www.sas.com/ and http://www.aacc.org/). Structuring of such information for particular disease states is crucial [27] and platforms for gene sets might improve understanding different biological data types to reach meaningful outputs [28]. Web mapping and power-grid including care for people on a medical device that depends on electricity may be included.

Software solutions for medical laboratories, that is, laboratory data management systems provide for a several decenny old experience on a local, intralaboratory scale. Data access strategies orient themselves on priority, storage options, and delivery methods. Long-term archival of data implies repeat migration from one media/storage environment to updated systems on regular schedule to prevent hacking.

## 5. Calibration, Steps, Hierarchy, and How to Scale

Laboratory data management and categorizing stand or fall with nomenclature of single analyses.

Whilst encryption is useful for privacy/intimacy, our golden age of surveillance likes coding as a (secrete) language system. The multilayer dimensionality of big data warehouses needs data-driven algorithms necessary to reach their basic goal: to translate big data into clinically useful evidence. Logistic regression models, Cox analysis, and Kaplan Meier curves can sort out analytes which would predict clinical evolution, for example, kidney-associated morbidity [30–32]; our own studies are being in line with researchers in Scandinavia. Platelet count and ICU survival, actually completely unrelated parameters, can be used for predictive modelling purpose [33]. One of the possibilities to unify assignments of terms for distinct analyses currently successful on an international level are the Logical Observation Identifiers Names and Codes (LOINC) which have been created under the auspices of the Regenstrief Inc. Institute, Indianapolis, IN, USA (http://search.loinc.org/) [34]. At present, the usage of LOINC codes remains subject to variations in the way they are used and semantic, taxonomic interoperability might turn out to be contradictory in some places [34]. Therefore, LOINC committees enforce detailed guidance on best practices for mapping from local to international LOINC codes and for using LOINC codes in data exchange [35]. Experiences using data warehouse produced collaboratively between academic medical centers and private practice throwing bridges to EHR do reveal potential to improve utilization of clinical pathology testing [36]. Thus, in the US, objective electronic laboratory reporting has now been promoted as a public health priority with two coding systems endorsed: LOINC for lab test orders and Systemized Nomenclature of Medicine-Clinical Terms (SNOMED CT) for test results, the former being in use more commonly [37]. LOINC is now* de rigueur* in France: Assistance Public des Hôpitaux de Paris, APHP, has created a biomedical observation dictionary mapped to LOINC which is bound to integrate this language into the entire biomedical production chain. Since its outset in 2010, participation of 120 laboratories including 50.000 codes now ensures interoperability in the entire French EHR system [38]. LOINC codes comprise categories to inform the data storage software on (i) the analyte, (ii) measured property, that is, enzyme activity or concentration, (iii) time (span) of sample collection, (iv) system used, for example, urine plasma serum, liquor, and (v) scale, that is, nominal and ordinal. One single analyte, for example, complement hemolytic activity activated through the classical pathway, CH50, comprises as much as 7 different LOINC codes (Table 1), depending on which starting material was employed for CH50 analysis.

Newborn screening, including such analyses with high-stake health implications necessitates rapid/effective communication between many people and organizations and increasingly depend on big data registries which can aggregate results from national programs [39] and help harmonize inherited metabolic disorder to an international level [40]. The March of Dimes recommends screening newborns for 29 conditions, such as phenylketonuria, hypothyroidism, galactosemia, and sickle-cell anemia (http://www.marchofdimes.com/). A prenatal screening program is taking shape and involves pregnancy-associated plasma protein A (PAPP-A) and pregnancy hormone hCG and pregnancies with hypertensive disorders might be detected early on using analysis of cell-free fetal DNA, cell-free total DNA, and biochemical markers [41].

A conjugate prior distribution belonging to the same parametric family may be chosen. Such a Bayes estimator for single analyses with their variance, confidence interval can be derived from a posterior distribution; the minimum square error also called squared error risk is defined by MSE = *E*[(*θ*(*x*) − *θ* ^2^)]. Some of the key features of a Bayesian analysis as a powerful package for molecular sequence variation have been delineated 10 years ago [42].

This novel task of doctors on scooping the right selection of relevant data requires a minimum understanding of what IT can and cannot do for the benefit of patient care (Table 2). The European Informatics Institute (EMBL-EBI) based on the consolidated Apache Lucene technology might inspire search engine development to direct scalable search paths towards medical diagnosis [43].

### 5.1. Big Data in Clinical Chemistry

The number of clinically relevant chemical analyses offered for diagnostic purpose at Swiss University Hospitals and private industry amounts up to roughly 180. Henceforward, such overseeable data built up during the last century currently expands to big data produced by automated workflow using intelligent robotics with throughputs of 3–15 million clinical chemistry assays/year. As learned from internet searches, Unilabs*™* processes 40.000 medical analyses/day and Synlab*™* offers > 4000 different analyses to its clients. A laboratory automate, for example, the Cobas machine (Roche Diagnostics, Rotkreuz, Switzerland) with its large panel of possible analyses, may serve as an example of ever growing lab service function. Competing industries, for example, Abbott (Abbott Diagnostics, Abbott Park, IL, USA), Siemens (Siemens Healthcare, Erlangen, Germany), Hitachi (Hitachi, Mountain View, USA), Capillarys Sebia (Paris, France), Kiestra (BD, Franklin Lakes, NJ, USA), Bruker (Bruker, Billerica, MA, USA), BioMérieux (Lyon, France), are continuously updating their offers in order to increase capacity, that is, number of analyses/time throughput. The analytes of the clinical chemistry lab section can be subdivided into provision of information for whole body pathology and into organ-specific lab assays. Selected groups of analyses are assembled as suggestion to clinicians in order to investigate single organs: endocrinology, liver function, gastroenterology, nephrology, vitamins. Each of these specialities of medicine sees its own lab definition increased in number of different analyses such as we have recently used cystatin C and its ratio to creatinine to improve significance of interpretation in kidney insufficiency [30]. The inflammation parameters in clinical chemistry, occasionally with biomarker status, are acute phase proteins, that is, CRP, serum amyloid A, fibrinogen, tryptase [44], haptoglobin, procalcitonin [45], interleukin-6, and again CRP used to classify disease stage of rheumatoid arthritis [20] and now even to estimate extent of fitness and senescence [11].

### 5.2. Big Data in Haematology

Two lines to enriching haematological patient findings emerged recently: (i) intelligent picture readings of blood and bone marrow films making possible telehaematology [46–48] and continuous flow analysis of single cells sorted according to their clonal origin. Picturing blood films does not lend itself to electronic storage in big data banks in contrast to findings coming out from forward and sideward scatter beamer flow cytometer cell analysers, such as Sysmex XE-5000 (Kobe, Japan), Abbot Sapphire (Abbott Diagnostics Division, Santa Clara, CA, USA), Siemens Advia (Siemens Healthcare, Erlangen, Germany), Beckman Coulter (Beckman Coulter Eurocenter, Geneva, Switzerland), and Amnis FlowSight (Seattle, WA, USA) [49].

Complete blood counts (CBC), hemostasis assays now forming part of Sysmex*™* machines, bone marrow, and progress in stem cell therapeutics are all prone to be integrated into big data banks.

The diagnostic value of both microscopic and automated neutrophil left-shift parameters as indicators of inflammatory disease is limited [50] but confirmed routine automated coagulation assays and pharmacomonitoring of many drugs with LC MS/MS machines are fit for integration into big data banks and miniaturization of assay principles is contributing to this trend [51].

Blood group typing, the classical way, (still) uses haemagglutination systems with monoclonal antibodies by and large on automated platforms [52]. Results from haemagglutination are now fully completed and soon will be replaced, at least in part, by genotyping procedures [53–56]. Multiple data release prone to be sections in big data registries are now being making precision medicine even more precise. In fact, inexpensive molecular typing of histoblood types paired with powerful bioinformatics has enabled mass-scale information but bears the risk that personalized red blood cell matching for transfusion becomes less precise [55]. Small- and large-order haemogram blocks for blood microarray technology can be used to more precisely delineate anti-ABH antibodies, a progress which will make solid organ transplantation across ABO barriers more successful thanks to using bioinformatics [14].

Stem cell transfusion and cord blood based therapy are making inclusion of HLA types and GWAS whole genome typing into registries which are based on big data informatics (http://www.hpscreg.eu/).

### 5.3. Big Data in Immunology

Analytical approaches of the immune system branches into cellular and humoral patient samples which are tested frequently (Figure 2). The order form of our institution lists up to 200 analyses offered to the clinician, not including the large field of tests to rule out allergic diseases. Care for patients suffering from allergic diseases recognizes the usefulness of a systemwide profiling approach, which associates big lab data with the biological approach to asthma and allergy [57]. Cellular immunological analysis overlaps with hematological tests of the myeloid compartment, but they make their own data box with lymphocyte subsets and their CD marker pattern. Close to 400 CD markers have been identified up to now and their number might grow.

**Figure 3:**
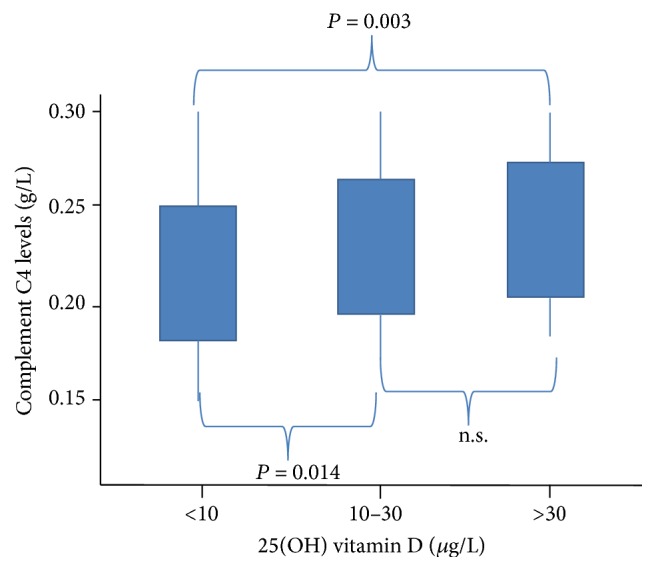
Complement component C4 levels depend on vitamin D sufficiency. With increasing vitamin D levels up to sufficiency (abscissa), the C4 serum levels statistically significantly increase in this cohort of healthy senior study participants > 60 years of age. From Sakem et al. 2013 [60]. A cohort of 1,470 healthy Swiss men and women, 60 years or older, were recruited for this study. A total of 179 subjects dropped out of the study because of elevated serum concentrations of C-reactive protein (>5 mg/L) making occult inflammation suspicious. 25(OH) vitamin D was measured using HPLC and levels were corroborated by parathyroid hormone measurements (not shown). The C4 levels were measured using immunonephelometry.

**Figure 4:**
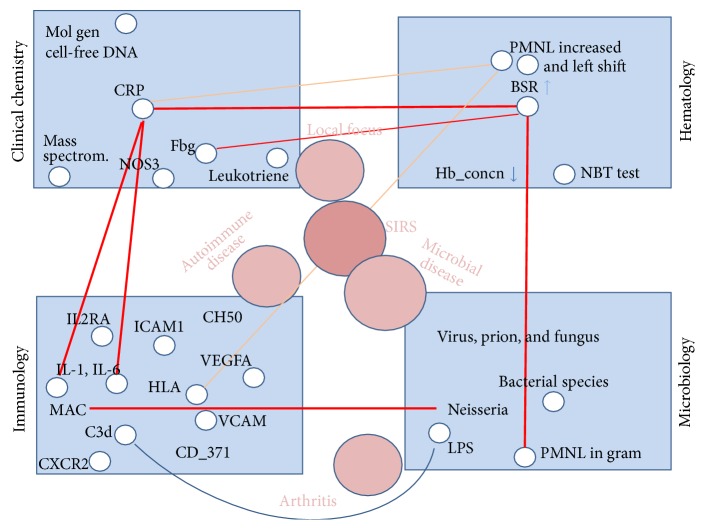
Analyses from different sections of the medical laboratory must be pulled together to establish evidence for inflammation. Similar to the display in Figure 2, the 4 major specialities, clinical chemistry, haematology, immunology, and microbiology, each encompass a whole array of inflammation markers. Networking across lab specialities obtrudes to pin down inflammatory disturbance. Complement analytics placed in the “immunology” box. Red bars connect strong evidence, while orange bars show medium evidence for denoted disease states. The insinuated disease states are placed arbitrarily.

Contribution of the complement system to big data is considerable; with three activation pathways ~48 proteins and their fragments, 9 protein complexes and ~12 receptors [58], measured w/v and/or by functional activity, the complement system contributes to warehouses on its own. Extraction of a selection of relevant data for patient care, data from data warehouses to which complement levels contribute involves interdisciplinary algorithm flow charts. These are focusing on diagnostic and therapeutic needs in precision medicine and so far are based on care for patients with immunological and microbial diseases. However, complement analysis should also be seen in perspective with other types of analyses. With the SENIORLAB study (ISRCTN registry number 53778569) we put w/v concentrations of C4 and C3 and immunoglobulin (Ig) levels into perspective with serum vitamin D levels and senescence. Immunoassays were used to quantitate C4 and C3 and Ig in 1470 apparently healthy subjects >60 yrs. Low levels of 25(OH)D were positively associated with IgG2 and C4 (the lower vitamin D, the lower C4, Figure 3) yet inversely related to levels of IgG1 and IgA and C3 [59, 60]. As can be seen in Table 1, a single one and the very same analysis can account for 7 different LOINC codes depending on the material in which measurements are made and depending on w/v versus functional performance measured. Acute phase complement proteins related to C-reactive protein (CRP) evolve in parallel during inflammatory states and are now known to play a role in type 2 diabetes, lipid metabolism, and atherosclerosis [61] (Figure 4).

These insights suggest that complement system-related algorithms destined to extract significant patient data from big data must be seen related to noncomplement analyses performed in the routine laboratory.

On the immune cell level, high-throughput sequencing has sparked information on TCR repertoire diversity informative on functional capacity of the adaptive immune system. Diagnostic applications have been limited to measuring inflammatory markers or identifying antibodies. Nothing but the diversity of *αβ* TCRs is mirrored by a receptors' dispersity based on different peptide-sequence which might reach > 1000 [62]. Given these large numbers, high-throughput sequencing is required to achieve sufficient sequencing depth to estimate clonal abundance. TCR *αβ* pairing can be assessed only at the single-cell level [63].

Big data warehouses will certainly have to make reference to the age group of study subjects. Thus, we have seen that IL-6 levels were lower and TNF-alpha reference intervals were higher in healthy newborns and toddlers than the adult reference intervals [64].

Biomarkers relating to particular disease states, for example, prostate-specific antigen (PSA) [65] or those of oxidized lipoproteins, genetically determined come to increase the size of big data in personalized medicine, their heritability being under scrutiny with twin pair studies [66].

Personal EHR health profiles captured by individuals themselves (e.g., from smart phones and wearable devices) will contribute to the next wave in big data—upload to the cloud and propagation across social networks make encryption prevent access to information by insurance companies, ransomware hackers, and state writ guardians [22].

### 5.4. Big Data in Medical Microbiology

Host, microbiomes, and pathogenic microbes are analyzable with a big data array of laboratory criteria making bioinformatics an indispensable pillar of big data in inflammation exploration. Bacterial, viral, fungal, and parasitic infections and diseases due to microbial toxins are among the most common and medically important causes of inflammation with different pathogens eliciting varied responses ranging from mild and short-term to severe and long-term [67]. Foreign bodies, catheters [68], splinters, sutures, and dirt may elicit inflammation and inflammation tissue and laboratory data may circumscribe hypersensitivity and autoimmune disease induced inflammation under the control of cytokines produced by T lymphocytes mainly. On hosts' side the predisposition to provide for a favourable environment for infectious agents, an array of receptor molecules (Fy a/b for malaria, CHO recognition on PMNL, fibronectin on catheters, Figure 4) can be appreciated to then enter BMLD (Biosafety in Microbiological and Biomedical Laboratories) banking. Nothing but the human gut containing a myriad of different bacteria and other microorganisms such as Archaea, viruses, and fungi, making the microbiome expand to big data sets, an enter-system [69]. On the side of microbes, the BMLD provides important information on the clinical presentation the infectious agent will cause if successfully attacking the host. As an example, the strain K 157 of* E. coli* is predictive of HUS and other strains of* E. coli*, such as O157:H7, O104:H4, O121, O26, O103, O111, O145, and O104:H21, produce potentially lethal toxins. Most* E. coli* are being innocuous or form part of the microbiome component. This is but one example of the enormous extent of data which microbiology occupies the space of a data warehouse. With the MALDI TOF system, fast typing has entered the practice since a decade [70] which facilitates updates of BMLD boxes. Virulence factors can be spotted in* Staphylococcus aureus* using whole genome sequencing combined with DNA microarray hybridization prone to increased big data informatics [23]. A large EHR data accumulation is in progress for diagnostic testing. For viral diseases as well laboratory assays differ between specimen analyses in acute disease and the assay approach used for specimens taken during convalesce. The current example here is guideline updates considering the ongoing Zika virus and other flavivirus epidemics (e.g., dengue, yellow fever, St. Louis encephalitis, and West Nile virus) which enforce usage of RT-PCR, also for chikungunya viruses. Proposed test algorithms start out with both molecular and antibody testing to minimize the risk for cross-reactivity vulnerable meandering (memorandum CDC) (http://www.cdc.gov/zika/pdfs/denvchikvzikv-testing-algorithm.pdf).

### 5.5. Big Data in Genetics

Whilst big genetic data validation overlaps and completes each of the preceding subchapters (Figure 2), genetic studies of many diseases are now allowing closer insights into human pathology. Next generation sequencing (NGS) creates challenges for validation of results. NGSs can be used to detect genetic anomalies of essentially any size scale, from SNPs to very large rearrangements; all of todays' genetic diagnostic tests could in principle be supplanted by NGS [71], including RNA analysis, because transcriptome (RNA-seq) sequencing is possible and now boosts with CRISP-Cas9 technology [72]. On the leading edge of a revolution in medicine the complete DNA sequence, properly encrypted, will become a permanent part of individual's EHR utilized by health care professionals to make decisions about drug prescriptions, diagnostics, and disease prevention [73]. Molecular geneticist validation reviews all variants called on a 10-gene panel with 30 variants per case without additional IT support, but it will be swamped when this would be have to be done for a 100-gene panel [17].

As an example phenotypes are now closely linkable to genetic findings and to the age of the patient, younger age at diagnosis associated with extensive/aggressive Crohn's disease and ulcerative colitis. Dissection of genotype-phenotype relations using Immunochip array designed to capture up to 200 different loci associated with common autoimmune diseases can be focused on NOD2, MHC, and MST1 3p21 to allow establishment of a genetic risk score and predictive modelling [74]. The genetic diseases encountered in medical practice are the tip of the iceberg, that is, those with less extreme genotypic glitches that permit full embryonic development and live birth. Genetic variants of complement genes, for example, CFIGly119Arg, such as recently evaluated in the EUGENDA cohort may be associated with age-related macular degeneration [75]. How many mutations remain hidden? With the determination of the complete sequence of the human genome, DNA imprints not only for disease but also for the gray zone between health and disease are under scrutiny. As an example, training of legasthenics could use genomic insights to improve its efficacy. As Dr. Collins puts it “it will take decades, if not centuries to understand the instructions of the genetic language, and, one might add, improve and make big data information more meaningful” [76].

## 6. Attempts to Constrain Big Data in Clinical Settings

Ransomware attacks hackers blocking hospital and private practice computers have now been reported here and there. Computer systems, including those needed for lab work, can be set out of function relatively easily which makes big data clouds vulnerable to an extent to which some workers keep copies on separate hardware aside; desktop virtualization systems, for example, Citrix*™*, are improved for hacker risk reduction and saving data apart on separate servers might do the rest to prevent big data hacking.

Two motive forces are thriving attempts to reduce the number of health parameters (i) to bring customers of the medical lab to a reasonable and patient driven block of analyses asked for [77] and (ii) to optimize financial sources doctors often ignoring the financial consequences of their test-ordering behavior.

Study results from attempts to reduce the number of ordered tests become known [78] and an expert panel may bring some good ideas but will never be able to brake medical revolution continuing to creep on us, especially in the field of laboratory analyses.

In conclusion, the current expansion of medical lab assay number expands big data warehouses. Multidisciplinary efforts are required to master the mighty offer of data and to make the information improve patient care.

## Figures and Tables

**Table 1:** Contribution of one single analysis, complement total hemolytic activity, CH50, to big data exemplified by LOINC coding. Seven different codes are attributed to the single CH50 analysis derived from difference in the long name, property, and the different body fluid systems in which CH50 can be measured.

LOINC code	Long name	Component	Property	System
55448-5	C total hemolytic CH50 in serum or plasma	C total hemolytic CH50	—	Ser/Plas
4511-2	C total hemolytic CH50 (units/volume) in body fluid	C total hemolytic CH50	ACnc	Body fluid
21218-3	C total hemolytic CH50 (units/volume) in cerebral spinal fluid	C total hemolytic CH50	ACnc	CSF
4532-8	C total hemolytic CH50 (units/volume) in serum or plasma	C total hemolytic CH50	ACnc	Ser/Plas
30131-7	C total hemolytic CH50 (mass/volume) in serum or plasma	C total hemolytic CH50	MCnc	Ser/Plas
48071-5	C total hemolytic CH50 (titer) in serum or plasma	C total hemolytic CH50	Titr	Ser/Plas
48496-4	C total hemolytic CH50 actual/normal in serum or plasma	C total hemolytic CH50 actual/normal	RelCCnc	Ser/Plas

**Table 2:** Big data in electronic health records (EHR). A 2016 update with special emphasis on medical laboratory medicine.

Advantages	Drawbacks	Measures	Ref
Real time health profile	Hacker friendliness	Encryption	[22]
Ubiquitous access to electronic health record EHR	Code readability not yet universal	National health offices regulation in progress	See http://www.medicalrecords.com/
Patients' own medical record	Patient-driven medical updates necessary	Transmit medical terminology to patient	Dragon Medical software
Patient record download by hospital W-LAN	Hacker friendliness	Limit time of accessibility	SOARIAN Health Archive (Switzerland) HIPAA Space (USA)
Distribution pattern of virulence of the same bacterial strain	Exchange of DNA: most strains have overlapping genome	(i) Update bioinformatic resource (ii) Use hybridization of identification	[23]
Postmarketing surveillance of medical devices	Criteria selection	LOINC*™* coding	[24]

## Abbreviations

Mol gen: Molecular genetic analyses
Fbg: Fibrinogen
NOS3: Nitric oxide synthase
PMNL: Polymorphonuclear neutrophilic leukocytes
Hb: Haemoglobin
NBT: Nitroblue tetrazolium
IL2RA: Interleukin-2 receptor subunit alpha
ICAM: Intercellular adhesion molecule
IL: Interleukin
HLA: Histocompatibility locus
CXCR2: C-X-C motif chemokine receptor 2
VEGFA: Vascular endothelial growth factor A
VCAM: Vascular cell adhesion molecule
LPS: Lipopolysaccharide
SIRS: Systemic inflammatory reaction syndrome
CD_371: CD marker currently coming up to 371 varieties
BSR: Blood sedimentation rate
MAC: Membrane attack complex.
